# Filgrastim Efficiency in Cats Naturally Infected with Feline Panleukopenia Virus

**DOI:** 10.3390/ani14243582

**Published:** 2024-12-11

**Authors:** Mihaela Anca Dascalu, Florentina Daraban Bocaneti, Octavian Soreanu, Paul Tutu, Andreea Cozma, Serban Morosan, Oana Tanase

**Affiliations:** 1 Department of Public Health, Faculty of Veterinary Medicine, “Ion Ionescu de la Brad” Iasi University of Life Sciences, Mihail Sadoveanu Alley, No. 8, 700489 Iasi, Romaniaoctaviansoreanu@yahoo.com (O.S.); tutu_paul@yahoo.com (P.T.); serban.morosan@iuls.ro (S.M.); tanase_oana@yahoo.com (O.T.); 2 Department of Exact Sciences, Faculty of Horticulture, “Ion Ionescu de la Brad” Iasi University of Life Sciences, Mihail Sadoveanu Alley, No. 3, 700490 Iasi, Romania; andreeapaulacozma@yahoo.com

**Keywords:** feline, filgrastim, hG-CSF, Feline panleukopenia virus, Zarzio

## Abstract

Feline Panleukopenia (FPL) infection is caused by feline panleukopenia virus (FPV), and it is considered one of the most severe cat’s infectious diseases. Since there is no specific antiviral treatment for FPL, the therapeutic protocol usually is focused on fluid therapy and supportive care. However, filgrastim, a granulocyte colony-stimulating factor (G-CSF) used in human medicine to treat neutropenia and leukopenia, has been lately used in treating FPV disease, providing promising results. During January 2022 and September 2024, twenty-two cats diagnosed with feline panleukopenia virus were subjected to filgrastim (Zarzio^®^, Sandoz, Kundl, Austria) administration at a dose of 6 µg/kg for 3 consecutive days. The 4th day was a break day, and in the 5th day, the complete blood count was repeated. White blood cells, lymphocytes, monocytes, neutrophils and eosinophils parameters improved after Zarzio^®^ administration, with a significant statistical difference (*p* < 0.01) when their values between day 1 (pre Zarzio^®^ administration) and day 5 (post Zarzio^®^ administration) were analysed. However, red blood cells, haemoglobin, haematocrit and platelets parameters registered a considerable reduction from day 1 to day 5 with a significant statistical difference (*p* < 0.01), considered as post-administration side effects. In our study, the survival rate following Zarzio^®^ administration was 100%, suggesting that the protocol involving three doses is effective in restoring the leukopenia and neutropenia.

## Simple Summary

Feline Panleukopenia (FPL) caused by feline panleukopenia virus (FPV), a DNA virus from genus *Protoparvovirus*, family *Parvoviridae*, is a highly contagious infectious disease of cats, often fatal, characterized by acute severe enteritis, vomiting, depression, dehydration and reduction in circulating white blood cell count. Given the low rate of survival, the lack of a specific antiviral drugs against FPV and the severe decrease in circulating white blood cell count resulting in immunosuppressed cat patients, over the years, several drugs were proposed and tested in order to find a cure. However, a promising human drug, represented by a granulocyte colony-stimulating factor (G-CSF), filgrastim, respectively, was tested and adapted in veterinary medicine therapy. The use of this hG-CSF in FPV cases from our study was demonstrated to be effective, providing evidence for its safe use. Human granulocyte colony stimulating factor represented by filgrastim should be regarded as a promising therapeutic option in cats diagnosed with FPL.

## 1. Introduction

Feline Panleukopenia (FPL) is a highly contagious infectious disease of cats, often fatal, characterized by acute severe enteritis, vomiting, depression, dehydration and reduction in circulating white blood cell count [1]. FPL is caused by feline panleukopenia virus (FPV), a DNA virus from genus *Protoparvovirus*, family *Parvoviridae* [2], known to be highly stable in the following environmental conditions: at room temperature it may survive up to one year, while outside it was reported to be viable up to 2 years [3]. Moreover, the virus is very resistant to trypsin, solvents, acids and bases [4]. The virus is mainly transmitted via faecal–oral route, although via utero infection and infection through fomites are possible [5].

The circulation of FPV in the feline population is maintained mainly by the stray cats. The most susceptible are kittens up to 12 months of age, with a rate of mortality ranging from 25 to 90% in acute infections and up to 100% in peracute infections [6,7,8]. However, in the last years, the incidence started to decrease, as the disease is well controlled by vaccination in many countries [9].

FPV presents an affinity for mitotically active cells, such as bone marrow (resulting in neutropenia, later anaemia and thrombocytopenia), lymphoid tissues (resulting in lymphopenia) and intestinal epithelia (resulting in severe diarrhoea and vomiting) [9,10]. Additionally, when the transmission via utero infection occurs, the virus targets the foetus which in turn may result in congenital defects, abortion, stillbirths and fading kittens. When the infection is occurring in the neonatal period, cerebellar hypoplasia with ataxia and tremors may result [3]. Moreover, retinal dysplasia, optic nerve hypoplasia or dysplasia were reported in kittens in the pre- or neonatal period [11].

Given the low rate of survival, the lack of a specific antiviral drugs against FPV [4] and the severe decrease in circulating white blood cell count resulting in immunosuppressed cat patients, over the years, several drugs were proposed and tested in order to find a cure. In this regard, recombinant feline interferon omega (rFeIFN) [12], the immunomodulatory drugs represented by the inactivated *Propionibacterium acnes* and lipopolysaccharide from *Escherichia coli* [13], F(ab’)2 fragment prepared from inactivated FPV-immunized horses [4], the inactivated parapox ovis strain D1701 (Zylexis) or a stabilized equine serum with neutralizing antibodies against FPV (Feliserin) [14] were proposed as therapy against FPL. A promising human drug, represented by a granulocyte colony-stimulating factor (G-CSF), filgrastim, respectively, was tested and adapted in the veterinary medicine therapy [15,16,17,18,19,20,21,22,23,24,25,26,27,28,29,30,31].

The colony-stimulating factors (CSFs) are divided into two main groups: G-CSF, including filgrastim, lenograstim, nartograstim and pegfilgrastim and granulocyte macrophage colony-stimulating factors (GM-CSFs), comprising sargramostin, molgramostim and regramostim [32]. The history of filgrastim started in 1984, when it was isolated for the first time from various human cells [33] such as macrophages, monocytes, endothelial cells or fibroblasts [34], after an appropriate stimulation, and continued with its purification and molecular cloning [35]. Given its promising results in human cancer therapy studies, the clinical trials’ started in the same year, culminating with its approval in 1991 in the therapy of cancer patients [36]. G-CSF is a myeloid factor that stimulates the growth of new blood cells [35] leading to an increase of the number of circulating neutrophils [37]. G-CSF acts on hematopoietic cells by binding to specific cell surface receptors, stimulating the proliferation‚ differentiation and maturation of neutrophil progenitors. In this concern the biological activity of filgrastim is similar to that of endogenous G-CSF. The amino acid sequence of filgrastim is identical to the endogenous G-CSF but is non-glycosylated and has an N-terminal methionine added in the sequence for expression in *Escherichia coli*, unlike endogenous G-CSF. In animals, filgrastim is rapidly distributed in high concentrations in the bone marrow, adrenal glands, kidneys and liver [38]. 

Filgrastim has various therapeutic uses, such as reduction of the incidence and duration of sequelae of neutropenia in symptomatic patients with congenital, cyclic or idiopathic neutropenia, management and prevention of infections and febrile neutropenia, consecutive to chemo or radiation therapy [38,39].

In veterinary medicine, a few studies were undertaken in order to analyse the efficiency of filgrastim in various species, such as cats [16,17,18,20,23,25,26,28,29,30], dogs [15,20,21,22,24,27,31,40], neonatal foals [19], lactating dairy cows [41], rabbits [42], alpacas [43], cetaceans such as belugas *(Delphinapterus leucas*), bottlenose dolphins (*Tursiops truncatus*), killer whales (*Orcinus orca*) and short-finned pilot whales (*Globicephala macrorhynchus*) [44], with or without various granulocyte disorders, represented by neutropenia of different origins, marrow stromal disorders and myelodysplastic syndrome/leukaemia [32,45]. Moreover, in feline patients with FPV infection, the result of the studies suggested that filgrastim may be used as a potential therapy [23,26,28,29,30]. In human patients, the filgrastim dose varies according to the pathology from 1 to 10 μg/kg [46]. In pets, different dosages were reported, such as 4.3 μg/kg [30], 5 μg/kg [26], 6 μg/kg [28,29] and 10 μg/kg [31], administered between 3 and 5 days.

The aim of the present study was the evaluation of hG-CSF (Zarzio^®^—filgrastim) efficiency in cats diagnosed with FPL induced by FPV.

## 2. Materials and Methods

### 2.1. Animals

During January 2022 and September 2024, 22 cats with suspicion of infectious diseases, represented by eight females and fourteen males, were presented at the Infectious Diseases Clinic, within Department of Public Health, Faculty of Veterinary Medicine of Iasi, Romania. A detailed history of each case, regarding the life style, the deworming, vaccination and neutered status, concurrent infections and clinical signs, observed by the owners or during the physical examination, was obtained (Table 1).

### 2.2. Ethical Approval

The animal study protocol was approved by the Ethics Committee of “Ion Ionescu de la Brad” Iasi University of Life Sciences, Faculty of Veterinary Medicine (protocol code: 0003/2021 and date of approval: 30 June 2021).

### 2.3. Physical Examination

A complete physical examination was performed for each case. This included the body temperature, heart and lung auscultation and rate, mucous membranes colour, examination of superficial lymph nodes, abdominal palpation, capillary refill time, skin turgor, extremity temperature, oral cavity, hair coat and skin.

### 2.4. Laboratory Tests

After the physical examination, to rule up a possible FPV infection, each cat was screened for FPV faecal antigen through a quantitative rapid immunochromatographic test performed with the Vcheck V200 apparatus or a qualitative immunochromatographic test (FPV Antigen Rapid Test Device—Faeces/Vomit, Rapid Labs, Colchester, UK), according to the manufacturer recommendations.

In this regard, a rectal swab from each cat was collected. Simultaneously, on blood collected from the jugular or cephalic vein, a complete blood count (CBC) using *VetScan HM5* Veterinary *Hematology Analyzer* (Abaxis, Inc., Union City, CA, USA) was performed for each cat. Considering the financial resources of the owners, blood biochemistry and cytology were possible only for a part of the cases.

### 2.5. Statistical Analysis

Statistical analysis was performed using SPSS IBM version 21 software for the *t*-test. A “*p*” value of < 0.01 was considered statistically significant for CBC parameters.

### 2.6. Therapeutic Protocol

Since there is no specific antiviral treatment for FPL, the therapeutic protocol usually is focused on fluid therapy and supportive care [47]. However, filgrastim, found as Neupogen^®^ (Amgen Inc., Thousand Oaks, CA, USA) and Zarzio^®^, has been lately used in treating FPV disease, providing promising results [26,28,29,30]. Therefore, FPV positive cats with panleukopenia confirmed by CBC were subjected to specific treatment, consisting of Zarzio^®^ administration.

#### Zarzio^®^ Administration Protocol

In this study, Zarzio^®^ (Sandoz, Kundl, Austria, syringe of 30 million units/0.5 mL, corresponding to 300 µg of filgrastim in 0.5 mL) was used. The dose administered, as previously reported by [28,29], was 6 µg/kg, subcutaneously, in the first 3 days of treatment, the 4th day was a break day and in the 5th day, the CBC was repeated in order to check if there is necessary a supplementary dose in the 5th and 6th day. All the cats were monitored for side effects. Given the normal range of the white blood cell (WBC) level, as confirmed by CBC analysis, the product was given for 3 days in all cases.

Since the patients are presented with lack of appetite, dehydration, vomiting and diarrhoea, appropriate fluid therapy and supportive care was provided.

## 3. Results

The cats included in this study aged between 3 months and 5.7 years old. Out of 22 cats, fourteen (63.63%) were represented by males and eight (36.37%) by females. During the study period, in 2022 there were recorded ten cases, in 2023 four cases and in 2024 eight cases.

Twenty of the cats were of European breed, while the other two were British Shorthair breed. Regarding the life style, seven cats were living exclusively indoors and seven were living exclusively outdoors, while the other eight had a mixed life style (indoor/outdoor). Except the two British shorthair cats, all the others were adopted from the street when they were kittens, without any history of deworming and vaccination. Concerning the vaccination status, seventeen cats had never been vaccinated and four received a vaccination shot but without booster, while one cat was vaccinated 5 days before presenting the clinical signs.

During physical examination, non-specific symptoms were noticed for the majority of the cats, such as lethargy, depression, lack of appetite and fever. Subsequently, during the hospitalization, gastrointestinal signs consisting of vomiting and watery, jelly-like diarrhoea (in some cases with streaks of blood) were noticed, which in turn leaded to severe dehydration, with a prolonged skin turgor and capillary refill time and pale mucous membranes. For ID cases 30645 and 30185, FPV disease was associated with the feline Calicivirus (FCV) leading to ulcers in the oral cavity, especially on the tongue, stomatitis, gingivitis and hypersalivation. Additionally, the ID case 22566 presented ataxia and tremors as a consequence of the nervous system infection induced by the virus.

All cases (22/22) tested positive for FPV faecal Ag by quantitative/qualitative immunochromatographic rapid tests. Further, the positive FPV cats with panleukopenia confirmed by CBC were subjected to Zarzio^®^ protocol administration.

The initial CBC analysis performed for each patient revealed leukopenia (22/22), lymphopenia (18/22), monocytopenia (20/22), neutropenia (20/22), eosinopenia (12/22) and basopenia (22/22). Concerning the RBC, six cats presented anaemia and two cats polycythaemias. Six of the cats showed low HGB, while one cat showed a high HGB. Eight cats showed low HCT and one high HCT. Microcytosis was observed in one case, low MCH in one case, high MCHC in three cases, low RDWc in one case and high RDWc in two cases. Thrombocytopenia was reported in nine cases and mean platelet volume in one case (Table 2).

Following Zarzio^®^ administration, CBC was repeated in the 5th day of the protocol for each patient. A notable improvement was achieved, with the patients fully recovering. Consecutively, fifteen out of twenty-two cats showed normal range values of WBC, and seven showed a slight to mild leukocytosis. No case of leukopenia persisted after Zarzio^®^ administration.

Twenty-one (21/22) cats presented normal range values of the lymphocytes and one (1/22) lymphopenia; 15/22 cats showed normal range values of the monocytes and 7/22 presented monocytosis; as regarding the neutrophils, 10/22 cats showed normal range values, while 12/22 presented neutrophilia; the values of eosinophils were in normal range for 21/22 cats, with one (1/22) case showing eosinophilia; 6/22 cats registered normal range values of the basophiles, while 16/22 presented basopenia. Regarding RBC, 7/22 of the cats presented normal range values, while the other 15 cats presented low values; HGB was in normal range in 8/22 cases and low for 14/22 cats; HCT showed normal values for 6/22 cats and low for 16/22 cats. MCV was found in normal range in all 22 cats; MCH was in normal range values in 21/22 cats, with one case low; MCHC had normal range values in 21/22 cats, with one case high value; RDWc was normal in 20/22 cats and in two (2/22) situations the values were above the normal range values. Concerning PLT count, normal values were found in only 3/22 cats, while 19/22 presented thrombocytopenia. MPV was in normal range for all the cats (Table 3). Detailed data regarding each case are presented in Appendix A, Table A1, Table A2 and Table A3.

**Figure 1:**
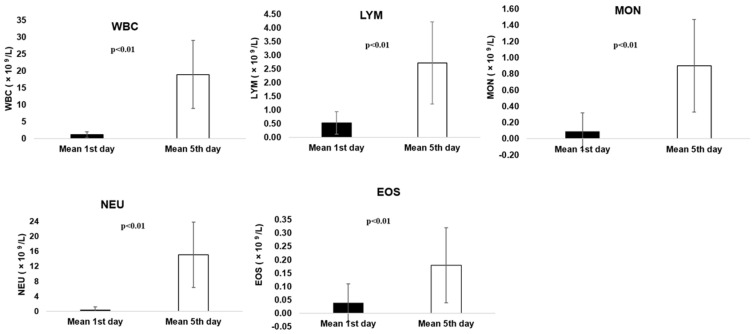
Pre and post Zarzio^®^ administration results for WBC, LYM, MON, NEU and EOS parameters.

A significant statistical difference (*p* < 0.01) was found between day 1 (pre Zarzio^®^ administration) and day 5 (post Zarzio^®^ administration) for WBC, LYM, MON, NEU and EOS parameters (Figure 1), showing significant improvement after Zarzio^®^ administration (Table A4).

**Figure 2:**
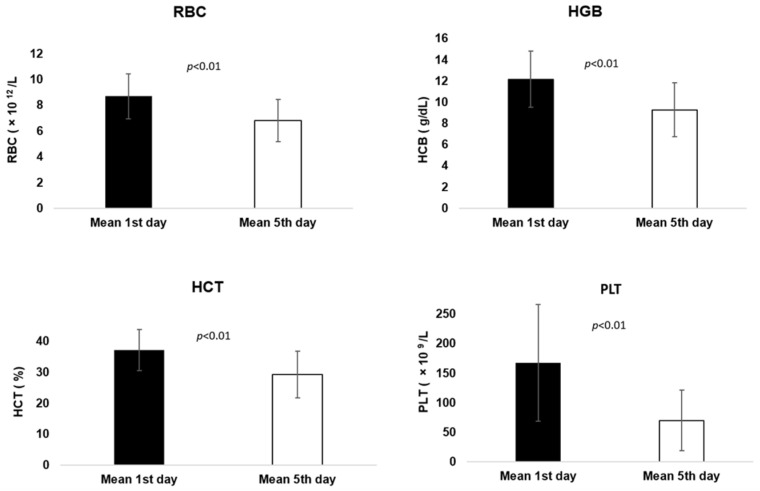
Pre and post Zarzio^®^ administration results for RBC, HGB, HCT and PLT parameters.

Additionally, a significant statistical difference (*p* < 0.01) was found for RBC, HGB, HCT and PLT parameters (Figure 2) after Zarzio^®^ administration, but on the contrary they registered a considerable decrease from day 1 to day 5, associated with post-administration side effects. Non-significant statistical analysis was registered for BAS, MCV, MCH, MCHC, RDWc and MPV (*p* = 0.144–0.915) (Table A4).

Beside FPV detection, other concomitant pathogens, represented by *Anaplasma platys* (ID case 22718 and 20784) and *Mycoplasma haemofelis* (ID case 21133), respectively, were diagnosed through blood cytology and treated accordingly.

All the cats involved in this study survived and fully recovered from FPL. The remission of gastrointestinal disorders was evident by the 3rd or 4th day after Zarzio^®^ administration, with reduced vomiting and diarrhoea frequency, with significant clinical improvement. The fluid therapy treatment and supportive care continued for up to 7 days, when they started to eat and drink voluntarily and became alert. After the discharge, the owners were advised to continue home medication (vitamins, iron supplements, pro- and prebiotics) administration.

## 4. Discussion

Human granulocyte colony-stimulating factor (hG-CSF) is a glycoprotein, a growth factor and a cytokine with a molecular mass between 18 and 20 kDa [48]. This glycoprotein is produced naturally by macrophages, epithelial cells or other immune cells and exits in two forms: one of 174 amino acids, considered the most active, abundant and used for drugs development and one of 177 amino acids, respectively. The difference between these two polypeptides consists in the presence or absence of three amino acids, but in both situations, they present authentic G-CSF activity, as it was demonstrated by expression studies. The gene of G-CSF was shown to be located on chromosome 17, locus q11.2–q12 [49]. Filgrastim has various medical purposes, from which the most important are to reduce the time to neutrophil recovery, the duration of neutropenia and to mobilize autologous hematopoietic progenitor cells into the peripheral blood [38].

Filgrastim is found under different commercial names such as Zarzio^®^ (Sandoz), Neupogen^®^ (Amgen Inc., Thousand Oaks, CA, USA), Leukokine^®^ (Inj 300, CJ Healthcare, Daegu, South Korea) or FRAVEN^®^ (Arven Ilac, İstanbul, Turkey). The aforementioned products contain the same concentration of filgrastim (300 µg/0.5 mL) and are obtained based on *E. coli* recombinant DNA technology. A slight variation between these products exists consisting of the excipient compounds list.

A few studies on human filgrastim in cats were undertaken in veterinary medicine, with promising results [23,26,28,29,30]. The first data regarding the efficiency of filgrastim in cats with FPV were published in the study of [23]. Twenty-six cats with severe neutropenia due to FPV received filgrastim (Neupogen^®^) for a 2-day trial period at a dose of 5 µg/kg, registering a significant overall increase of neutrophil granulocytes. This study was the starting point for further research on this topic. Therefore, a few years later, ref. [26] published a study on filgrastim (Neupogen^®^) use in seven cases diagnosed with FPL. The cats that were aged between 2 months and 3 years received filgrastim at a dose of 5 µg/kg, daily, subcutaneously, for 5 days. The last CBC performed, at the 5th day, revealed normal leucocyte and neutrophil values for the five cats, except one case that showed leukocytosis and neutrophilia. One cat did not survive after the first dose of filgrastim, after it developed a systemic inflammatory response syndrome. A confirmatory study of [28] on testing Neupogen^®^ in FPV cats showed a 90% (10/11) survival rate, concluding the efficiency of the product. Recently, a study performed by [30] on five cats with FPL showed a significantly improvement after filgrastim (Neupogen^®^) administration. The product was given in the first 3 days, respectively, in the 5th and 6th day, at a dose of 4.3 µg/kg, daily, subcutaneously. Four out of five cats survived and showed a complete resolution of leukopenia and neutropenia and a rapid clinical recovery.

In 2023, ref. [29] tested a human filgrastim (FRAVEN^®^, Arven Ilac, Turkey) on 31 cats, ages 2 to 24 months old, diagnosed with FPL. The product was given 3 days consecutively at a dose of 6 µg/kg, daily, subcutaneously. In the 4th day, a CBC for control was performed. A supplementary dose was given in the 5th and the 6th day only for the cats whose blood levels did not show improvement. The survival ratio in the filgrastim group was 74.49%, while in the control group, 58.82%, concluding that filgrastim is a promising solution for a successful treatment of FPL.

To the authors’ best knowledge, this study is the first to test Zarzio^®^ on cats diagnosed with FPL. After Zarzio^®^ administration, WBC normalized values were seen in 15/22 (68.19%) cats, while for the other 7/22 (31.81%), leukocytosis was observed. Leukocytosis identified in 7/22 cats and neutrophilia in 12/22 cats from the present study are in agreement with other publications [17,18,25,26]. In the study of [26], 4/5 positive FPL cats showed leukocytosis and neutrophilia after filgrastim administration, where the increased level of the neutrophils were assigned to the exogenous growth factors [26].

Additionally, filgrastim was also tested on healthy cats in order to check how the product can influence haematopoiesis [17,18]. In the study published by [17], different dosages of filgrastim were administered in two healthy cats for 21 days, resulting in significant increase of neutrophils. On the other side, ref. [18] tested a recombinant canine G-CSF in five healthy cats for 42 days in order to confirm if the product can be effective and safe to use in felines. In this case, neutrophilia was observed in all cats, considered to be a cause of ”release phenomenon”, where the neutrophils are released into the blood stream from the marginal granulocytic pool, but also a cause of an increased myeloid differentiation with stimulation of myeloid progenitors cells [18], represented by monocytes, tissue macrophages, granulocytes and dendritic cells [50].

Yamamoto et al. 2002 [25] succeeded in obtaining a feline G-CSF, expressed in *Escherichia coli*, for practical use purposes, and it was evaluated in comparison with a human G-CSF. In this regard, two cats were injected with a low level of purified feline G-CSF and another two with human G-CSF for two days, using different dosages, showing an increased level of the neutrophils. When the products were given daily for 11 days, a significant increase of the neutrophils, monocytes and lymphocytes were seen. After administration, the modified values started to decrease immediately after stopping the G-CSF injections. In all the aforementioned studies, the level of leucocytes, neutrophils, monocytes and lymphocytes were normalized after filgrastim administration stopped, suggesting its contribution in influencing the normal haematopoiesis process.

In our study, the blood parameters represented by WBC, LYM, MON, NEU and EOS showed a significant statistical difference (*p* < 0.01) between day 1 (pre Zarzio^®^ administration) and day 5 (post Zarzio^®^ administration) suggesting the Zarzio^®^ administration contributed to blood parameters’ improvement, as shown also by [23,26,29,30]. On the contrary, RBC, HGB, HCT and PLT parameters registered considerably low values from day 1 to day 5, a fact confirmed by the statistical analysis where a significant difference (*p* < 0.01) was noted, as it was shown by [37] for PLT parameter. Additionally, 9/22 (40.90%) cats presented thrombocytopenia prior to Zarzio^®^ administration. In the 5th day, beside the initially nine cats, another eleven cats (90.90%) developed slight to medium thrombocytopenia, considered to be induced by Zarzio^®^. Following filgrastim administration, various side effects may result, consisting of the following: leukocytosis, neutrophilia, thrombocytopenia or anaemia [17,18,25,39]. However, thrombocytopenia is one of the most common side effect reported after filgrastim administration in human patients, as shown by several studies [46,51,52,53,54,55].

Although the mechanism of G-CSF induced thrombocytopenia has not been fully elucidated, different hypotheses have been suggested. Among them, an increased platelet consumption can be a consequence of splenomegaly leading to PLT depletion from the peripheral blood or because of reticuloendothelial system activation [53,54]. In order to confirm this hypothesis, Takamatsu et al. 2007 [54] tested the G-CSF on normal and splenectomised mice, and PLT counts were measured during the treatment. It was shown that a short term (7 days) administration of G-CSF resulted in severe platelet reduction, while a long term (15 days) administration does not induce thrombocytopenia in transgenic mice. The study concluded that the PLT count reduction is dose-dependent and is considered to be a transient event with spontaneous improvement after the product is stopped.

Since in normal haematopoiesis, the platelets are produced from megakaryocytes, ref. [54] also analysed the number of cells in the bone marrow and spleen, respectively, in mice treated with G-CSF, concluding that a spontaneous improvement of G-CSF induced thrombocytopenia is not related to the number of megakaryocytes in the bone marrow. However, a recent study published by [56], demonstrated that G-CSF inhibits the differentiation of myeloid progenitors into megakaryocyte erythroid progenitors leading to a decreased platelet production.

Regarding the hG-CSF side effects in felines, scarce data are available. However, ref. [37] reported a transient thrombocytopenia following hG-CSF therapy in a Russian blue breed cat diagnosed with idiopathic epilepsy. The cat developed leukopenia and neutropenia, and after 5 months the treatment against epilepsy was stopped, while the other CBC parameters results were in range. To address this issue, filgrastim (Leukokine^®^ Inj 300, CJ Healthcare, South Korea) was administered. Two weeks post filgrastim administration, the cat developed severe thrombocytopenia, concluding that this drug was most likely the cause. As in human patients, where the reported thrombocytopenia spontaneously resolved, no treatment was initiated for the cat patient, and two weeks after, the animal platelets were within the reference interval.

Anaemia was another side effect to Zarzio^®^ administration noticed in our study. In prior Zarzio^®^ administration, 6/22 (27.27%) cases presented anaemia, while post Zarzio^®^ treatment 15/22 (68.19%) cats developed anaemia. This side effect was reported in human patients diagnosed with breast cancer, where the G-CSF administration produced a significant reduction in mean haemoglobin concentration and anaemia, progressively worsening with increasing dosage of the product [57], also mentioned by the manufacturer [39]. No reports from veterinary medicine were found. Normally, anaemia is observed in cats with FPV and is considered to be a consequence of bone marrow depression, immunosuppression, inflammatory condition or chronicity of the disease [58]. As stated by [10], non-severe anaemia could be masked by dehydration and could be visible only when the intestinal blood loss is severe. In our study, the increased number of cats with anaemia, could be attributed to both the virus effect on bone marrow and Zarzio^®^ administration. Regarding the other blood parameters, represented by BAS, MCV, MCH, MCHC, RDWc and MPV, no differences were noted, as confirmed by statistical analysis (*p* = 0.144–0.915).

In the present study, the main and consistent clinical signs observed in cats were the lack of appetite (20/22), lethargy (19/22) and fever (12/22). Subsequently, the cats developed other clinical signs, such as vomiting (6/22), diarrhoea (10/22), pale apparent mucous membranes (4/22), dehydration (11/22), ataxia and tremors (1/22), lateral decubitus (1/22) increased submandibular lymph nodes (1/22) and hypothermia (1/22). Non-specific signs, such as tongue ulcers (2/22), gingivitis (1/22), purulent nasal discharge (1/22) and noisy breathing (1/22), considered secondary complications. It should be mentioned that two of the cats diagnosed with FPL were spayed 2 weeks before getting infected with FPV, without any vaccine dose administration prior surgery, leading to a decrease immunity and favouring an infectious disease’s appearance.

Following the conventional treatment and because of the disorder severity, the survival ratio in cats infected with FPV was reported to vary between 11.2% and 57.1% [28,59,60,61,62,63].

The use of Zarzio^®^ along with the fluid therapy and supportive care treatment lead to 100% survival rate of the cats included in the present study. Consistent combined treatments are required in order to treat the severe clinical signs induced by FPV.

## 5. Conclusions

In our study, the rate of survival was 100% following Zarzio^®^ administration, demonstrating that the protocol involving three doses is effective in restoring the leukopenia and neutropenia, providing evidence for its safe use. It should be regarded as a promising therapeutic option. The administration in feline patients may result in side effects commonly consisting of thrombocytopenia and anaemia. Future studies on the effect of short or long term administration of hG-CSF on platelet counts should be investigated.

## Figures and Tables

**Table 1:** Characteristics of the samples included in the study.

No.	Species	Breed	Year of Sample Collection	ID Case	Age (m/y)	Gender	Life Style	Vaccination Status	Clinical Signs	Rapid Immuno- Chromatographic Test		Evolution
										Result	* COI	
1	Cat (*Felis catus*)	European	September 2024	33669	3 months	M	Outdoor	No	Lack of appetite, lethargy, fever, diarrhoea, dehydration, direct contact with 2 cats that died from FPV.	Positive	38.82	Recovered
2		European	June 2024	33008	3 months	F	Indoor and outdoor	Yes (5 days before getting sick)	Lack of appetite, lethargy.	Positive	3.53	Recovered
3		European	June 2024	32876	2 years	M	Indoor and outdoor	No	Lack of appetite, lethargy, fever, ulcers on the tongue.	Positive	40.71	Recovered
4		European	June 2024	27478	1 year	M	Indoor	No	Lack of appetite, lethargy, fever, vomiting, diarrhoea, dehydration, cystitis.	Positive	5.00	Recovered
5		European	April 2024	32199	3 years	M	Indoor and outdoor	No	Lack of appetite, lethargy.	Positive	12.09	Recovered
6		European	March 2024	31846	6 months	F	Indoor	No	Lack of appetite, lethargy, fever, diarrhoea, dehydration.	Positive	28.72	Recovered
7		European	February 2024	31451	8 months	F	Indoor and outdoor	No	Lack of appetite, lethargy, diarrhoea, dehydration, abscess on the abdomen and plague on the anterior left limb.	Positive	2.80	Recovered
8		European	January 2024	30918	9.5 months	M	Outdoor	No	Lateral decubitus, lethargy, dehydration, pale mucous membranes.	Positive	2.6	Recovered
9		European	December 2023	30645	1.6 years	M	Indoor	No	Lack of appetite, lethargy, fever, vomiting, diarrhoea with streaks of blood, dehydration, gingivitis.	Positive	28.8	Recovered
10		European	November 2023	30185	1.4 years	M	Indoor	Yes, but not in schedule	Noisy breathing, purulent nasal discharge, ulcers on the tongue.	Positive	1.01	Recovered
11		European	September 2023	29426	1.2 years	F	Indoor and outdoor	No	Lack of appetite, lethargy, pale mucous membranes, direct contact with 2 cats that died recently from FPV.	Positive	1.16	Recovered
12		European	September 2023	29319	1 years	M	Outdoor	No	Lack of appetite, lethargy, diarrhoea, dehydration, direct contact with a cat that died recently from FPV.	Positive	Qualitative test	Recovered
13		European	December 2022	25382	1.10 years	F	Outdoor	No	Lack of appetite, lethargy, fever, direct contact with a cat that died recently.	Positive	3.16	Recovered
14		European	November 2022	25296	2.2 years	F	Outdoor	No	Lack of appetite, lethargy, pale mucous membranes, fever, direct contact with a cat that died recently from FPV.	Positive	Qualitative test	Recovered
15		European	November 2022	25213	2.2 years	M	Outdoor	No	Lack of appetite, lethargy, pale mucous membranes, vomiting, watery diarrhoea with streaks of blood, dehydration, submandibular lymph nodes increased in volume, hypothermia.	Positive	Qualitative test	Recovered
16		British Shorthair	October 2022	24766	7 months	M	Indoor	Yes, but not in schedule	Lethargy, fever, vomiting, diarrhoea, dehydration.	Positive	1.8	Recovered
17		European	June 2022	22718	2.11 years	M	Indoor	Yes, but not in schedule	Lack of appetite, lethargy.	Positive	12.54	Recovered
18		European	June 2022	22566	11 months	F	Outdoor	No	Spayed 10 days ago, lack of appetite, lethargy, fever, ataxia on the posterior limbs and tremors.	Positive	21.33	Recovered
19		European	March 2022	21283	1 year	F	Indoor and outdoor	No	Lack of appetite, lethargy, fever, spayed 1 week and a half ago.	Positive	Qualitative test	Recovered
20		European	March 2022	21133	3.3 years	M	Indoor and outdoor	No	Lack of appetite, lethargy, fever, vomiting, diarrhoea, dehydration.	Positive	Qualitative test	Recovered
21		European	March 2022	20784	1.7 years	M	Indoor and outdoor	No	Lack of appetite, lethargy, fever, plague at the left posterior leg.	Positive	Qualitative test	Recovered
22		British Shorthair	February 2022	20352	5.7 years	M	Indoor	Yes, but not in schedule	Lack of appetite, lethargy, vomiting, diarrhoea, dehydration.	Positive	Qualitative test	Recovered

* COI: cut-off index value (if the COI value is ≥1, the sample is considered positive).

**Table 2:** Cell blood count (CBC) findings pre Zarzio^®^ administration in cats.

No.	Parameter	Blood Condition	Number of Cats with FPL					Reference Ranges
			2022 (n = 10)	2023 (n = 4)	2024 (n = 8)	Total 2022–2024 (n = 22)		
						Total	%	
1	WBC	Leukopenia	10	4	8	22	100	3.5–20.7 × 10^9^/L
2	LYM	Lymphopenia	8	4	6	18	81.81	0.83–9.1 × 10^9^/L
3	MON	Monocytopenia	10	4	6	20	90.90	0.09–1.21 × 10^9^/L
4	NEU	Neutropenia	10	3	7	20	90.90	1.63–13.37 × 10^9^/L
5	EOS	Eosinopenia	7	2	3	12	54.54	0.02–0.49 × 10^9^/L
6	BAS	Basopenia	10	4	8	22	100	0–0.2 × 10^9^/L
7	LYM%	Lymphopenia	0	1	1	2	9.09	0–100%
8	MON%	Monocytopenia	0	1	1	2	9.09	0–100%
9	NEU%	Neutropenia	0	1	1	2	9.09	0–100%
10	EOS%	Eosinopenia	2	2	3	7	31.81	0–100%
11	BAS%	Basopenia	8	3	4	15	68.18	0–100%
12	RBC	Anaemia (low RBC)	2	2	2	6	27.27	7.7–12.8 × 10^12^/L
		Polycythaemia (high RBC)	1	1	0	2	9.09	
13	HGB	↓ HGB	1	1	4	6	27.27	10–17 g/dl
		↑HGB	1	0	0	1	4.54	
14	HCT	↓ HCT	3	2	3	8	36.36	33.7–55.4%
		↑ HCT	1	0	0	1	4.54	
15	MCV	Mycrocitosis	0	1	0	1	4.54	35–52 fl
16	MCH	↓ MCH	0	1	0	1	4.54	10–16.9 pg
		↑ MCH	0	0	0	0	0	
17	MCHC	↓ MCHC	0	0	0	0	0	27–35 g/dl
		↑ MCHC	2	1	0	3	13.63	
18	RDWc	↓ RDWc	0	1	0	1	4.54	18.3–24.1%
		↑ RDWc	0	0	2	2	9.09	
19	PLT	Thrombocytopenia	3	1	5	9	40.90	125–618 × 10^9^/l
20	MPV	↓ MPV	1	0	0	1	4.54	8.6–14.9 fl
		↑ MPV	0	0	0	0	0	

WBC: white blood cells, LYM: lymphocytes, MON: monocytes, NEU: neutrophils, EOS: eosinophils, BAS: basophils, RBC: red blood cells, HGB: haemoglobin, HCT: haematocrit, MCV: mean corpuscular volume, MCH: mean corpuscular haemoglobin, MCHC: Mean corpuscular haemoglobin concentration, RDWc: Red blood cell distribution width, PLT: platelets, MPV: mean platelet volume, ↓: low, ↑: high.

**Table 3:** Cell blood count (CBC) findings post Zarzio^®^ administration in cats.

No.	Parameter	Blood Condition	Number of Cats with FPL					Reference Ranges
			2022 (n = 10)	2023 (n = 4)	2024 (n = 8)	Total 2022–2024 (n = 22)		
						Total	%	
1	WBC	In range	7	2	6	15	68.19	3.5–20.7 × 10^9^/L
		Leukocytosis	3	2	2	7	31.81	
2	LYM	In range	10	4	7	21	95.46	0.83–9.1 × 10^9^/L
		Lymphopenia	0	0	1	1	4.54	
3	MON	In range	6	3	6	15	68.19	0.09–1.21 × 10^9^/L
		Monocytosis	4	1	2	7	31.81	
4	NEU	In range	4	2	4	10	45.46	1.63–13.37 × 10^9^/L
		Neutrophilia	6	2	4	12	54.54	
5	EOS	In range	10	3	8	21	95.46	0.02–0.49 × 10^9^/L
		Eosinophilia	0	1	0	1	4.54	
6	BAS	In range	2	3	1	6	27.28	0–0.2 × 10^9^/L
		Basopenia	8	1	7	16	72.72	
7	LYM%	In range	10	4	8	22	100	0–100%
8	MON%	In range	10	4	8	22	100	0–100%
9	NEU%	In range	10	4	8	22	100	0–100%
10	EOS%	In range	10	4	8	22	100	0–100%
11	BAS%	In range	2	1	0	3	13.63	0–100%
		Basopenia	8	3	8	19	86.37	
12	RBC	In range	4	1	2	7	31.81	7.7–12.8 × 10^12^/L
		Anaemia (low RBC)	6	3	6	15	68.19	
13	HGB	In range	3	1	4	8	36.37	10–17 g/dL
		↓ HGB	7	3	4	14	63.63	
14	HCT	In range	3	1	2	6	27.28	33.7–55.4%
		↓ HCT	7	3	6	16	72.72	
15	MCV	In range	10	4	8	22	100	35–52 fL
16	MCH	In range	9	4	8	21	95.46	10–16.9 pg
		↓ MCH	1	0	0	1	4.54	
17	MCHC	In range	10	3	8	21	95.46	27–35 g/dL
		↑ MCHC	0	1	0	1	4.54	
18	RDWc	In range	10	4	6	20	90.90	18.3–24.1%
		↑ RDWc	0	0	2	2	9.10	
19	PLT	In range	1	1	0	2	9.10	125–618 × 10^9^/L
		Thrombocytopenia	9	3	8	20	90.90	
20	MPV	In range	10	4	8	22	100	8.6–14.9 fL

WBC: white blood cells, LYM: lymphocytes, MON: monocytes, NEU: neutrophils, EOS: eosinophils, BAS: basophils, RBC: red blood cells, HGB: haemoglobin, HCT: haematocrit, MCV: mean corpuscular volume, MCH: mean corpuscular haemoglobin, MCHC: Mean corpuscular haemoglobin concentration, RDWc: Red blood cell distribution width, PLT: platelets, MPV: mean platelet volume, ↓: low, ↑: high.

## Data Availability

The original contributions presented in this study are included in the article. Further inquiries can be directed to the corresponding author.

## Appendix A

**Table A1:** Comparison of haematological parameters pre and post Zarzio^®^ administration in cats from 2022.

No.	Parameter	Pre and Post Filgrastim Administration	CAT ID SAMPLE										
			25382	25296	25213	24766	22718	22566	21283	21133	20784	20352	Reference Range
1	WBC	Day 1st	0.77	1.24	0.41	0.81	1.52	1.11	0.42	2.55	1.08	0.48	3.5–20.7 × 10^9^/L
		Day 5th	8.77	18.29	32.27	16.13	29.85	20.63	28.05	13.06	11.5	16.86	
2	LYM	Day 1st	0.6	0.76	0.3	0.56	0.8	0.96	0.28	1.73	0.73	0.38	0.83–9.1 × 10^9^/L
		Day 5th	1.59	3.27	3.55	4.04	2.42	3.09	2.46	1.27	1.89	1.78	
3	MON	Day 1st	0.05	0.05	0.02	0.02	0.08	0.03	0	0.09	0.05	0	0.09–1.21 × 10^9^/L
		Day 5th	0.61	1.47	1.81	0.73	1.32	1.16	1.5	1.06	0.78	0.1	
4	NEU	Day 1st	0.11	0.39	0.08	0.02	0.63	0.12	0.14	0.71	0.3	0.02	1.63–13.37 × 10^9^/L
		Day 5th	6.47	13.42	26.62	11.32	26.04	16.17	23.9	10.52	8.79	14.52	
5	EOS	Day 1st	0.02	0.04	0	0.2	0.01	0	0	0.02	0.01	0	0.02–0.49 × 10^9^/L
		Day 5th	0.1	0.13	0.3	0.03	0.07	0.22	0.17	0.21	0.03	0.46	
6	BAS	Day 1st	0	0	0	0.01	0	0	0	0	0	0	0–0.2 × 10^9^/L
		Day 5th	0	0	0	0	0	0	0	0	0	0.01	
7	LYM%	Day 1st	77.5	61.2	73.7	69.4	52.5	86.5	65.9	67.8	67	93.7	0–100%
		Day 5th	18.2	17.9	11	25.1	8.1	15	8.8	9.7	16.4	10.6	
8	MON%	Day 1st	6	3.7	5.8	3	5	2.8	0.4	3.5	4.5	1	0–100%
		Day 5th	6.9	8	5.6	4.5	4.4	5.6	5.4	8.1	6.8	0.6	
9	NEU%	Day 1st	14.2	31.9	20.5	2	41.6	10.6	33.6	27.9	27.6	5.3	0–100%
		Day 5th	73.8	73.4	82.5	70.2	87.2	78.3	85.2	80.6	76.5	86.1	
10	EOS%	Day 1st	2.3	3.1	0	24.6	0.9	0.1	0	0.8	0.8	0	0–100%
		Day 5th	1.1	0.7	0.9	0.03	0.2	1.1	0.6	1.6	0.3	2.7	
11	BAS%	Day 1st	0	0.1	0	1	0.1	0	0	0	0	0	0–100%
		Day 5th	0	0	0	0	0	0	0	0	0	0	
12	RBC	Day 1st	6.68	7.51	9.38	10.8	10.42	8.41	7.8	11.7	7.96	11.63	7.7–12.8 × 10^12^/L
		Day 5th	5.18	5.6	2.56	7.90	9.78	5.53	6.46	8.66	7.06	7.32	
13	HGB	Day 1st	8.9	11.2	13.5	16.8	16.3	11.9	11.5	15.7	10.6	15.6	10–17 g/dL
		Day 5th	6.5	8.3	3.3	11.6	15.1	7.7	8.7	11.6	9.9	8.8	
14	HCT	Day 1st	30.8	33.26	40.06	49.1	47.31	33.37	32.76	46.38	34.42	46.1	33.7–55.4%
		Day 5th	22.77	25.21	10.24	34.70	43.92	22.13	27.06	35.74	30.65	28.27	
15	MCV	Day 1st	46	44	14.4	45	45	40	42	41	43	40	35–52 fL
		Day 5th	44	45	40	44	45	40	42	41	43	39	
16	MCH	Day 1st	13.3	14.9	14.4	15.6	15.7	14.2	14.8	13.8	13.3	13.4	10–16.9 pg
		Day 5th	12.5	14.9	12.9	14.7	15.5	13.9	13.4	13.4	14.1	12.1	
17	MCHC	Day 1st	28.8	33.7	33.8	34.4	34.5	35.7	35.1	33.8	30.8	33.9	27–35 g/dL
		Day 5th	28.5	33	32.3	33.5	34.4	34.8	32.1	32.4	32.4	31.3	
18	RDWc	Day 1st	19	20.6	22.1	21.9	21.6	21	20.8	22	20.4	22.5	18.3–24.1%
		Day 5th	19.4	20.3	20.9	20	20.6	20.2	20.5	20.5	20.7	20.7	
19	PLT	Day 1st	86	141	202	236	402	149	67	259	109	314	125–618 × 10^9^/L
		Day 5th	26	73	12	23	227	36	43	96	55	80	
20	MPV	Day 1st	12.6	11.6	10.1	12.8	12.2	10.1	12.3	10.7	12.3	10.8	8.6–14.9 fL
		Day 5th	12.6	12.2	8.6	6.8	12.1	10.6	12	11.4	12.3	10.6	

WBC: white blood cells, LYM: lymphocytes, MON: monocytes, NEU: neutrophils, EOS: eosinophils, BAS: basophils, RBC: red blood cells, HGB: haemoglobin, HCT: haematocrit, MCV: mean corpuscular volume, MCH: mean corpuscular haemoglobin, MCHC: Mean corpuscular haemoglobin concentration, RDWc: Red blood cell distribution width, PLT: platelets, MPV: mean platelet volume.

**Table A2:** Comparison of haematological parameters pre and post Zarzio^®^ administration in cats from 2023.

No.	Parameter	Pre and Post Filgrastim Administration	CAT ID SAMPLE				Reference Range
			30645	30185	29426	29319	
1	WBC	Day 1st	2.28	3.07	0.37	0.66	3.5–20.7 × 10^9^/L
		Day 5th	12.78	28.52	12.23	45.35	
2	LYM	Day 1st	0.35	0.33	0.36	0	0.83–9.1 × 10^9^/L
		Day 5th	1.88	3.98	4.45	7.33	
3	MON	Day 1st	0.12	0.02	0	0	0.09–1.21 × 10^9^/L
		Day 5th	0.16	0.95	0.88	2.29	
4	NEU	Day 1st	1.78	2.63	0.01	0	1.63–13.37 × 10^9^/L
		Day 5th	10.3	23.35	6.83	35.19	
5	EOS	Day 1st	0.02	0.09	0	0	0.02–0.49 × 10^9^/L
		Day 5th	0.42	0.24	0.07	0.52	
6	BAS	Day 1st	0	0	0	0	0–0.2 × 10^9^/L
		Day 5th	0.01	0.01	0	0.01	
7	LYM%	Day 1st	15.6	10.8	97.2	0	0–100%
		Day 5th	14.7	13.9	36.3	16.2	
8	MON%	Day 1st	5.4	0.7	1	0	0–100%
		Day 5th	1.3	3.3	7.2	5.1	
9	NEU%	Day 1st	78	85.7	1.8	0	0–100%
		Day 5th	80.6	81.9	55.9	77.6	
10	EOS%	Day 1st	0.9	2.8	0	0	0–100%
		Day 5th	3.3	0.24	0.6	1.2	
11	BAS%	Day 1st	0.2	0	0	0	0–100%
		Day 5th	0.1	0.01	0	0	
12	RBC	Day 1st	12.38	6.63	8.87	7.19	7.7–12.8 × 10^12^/L
		Day 5th	8.94	6.92	4.72	4.73	
13	HGB	Day 1st	16.6	8.7	10.6	12.1	10–17 g/dL
		Day 5th	11.1	9.3	5.3	7.9	
14	HCT	Day 1st	49.25	29.26	35.1	33.01	33.7–55.4%
		Day 5th	38.2	31.39	18.79	21.79	
15	MCV	Day 1st	40	44	40	46	35–52 fL
		Day 5th	43	45	40	46	
16	MCH	Day 1st	13.4	13.2	11.9	16.8	10–16.9 pg
		Day 5th	12.4	13.5	11.1	16.6	
17	MCHC	Day 1st	33.6	29.9	30.1	36.5	27–35 g/dL
		Day 5th	29	29.6	28	36.1	
18	RDWc	Day 1st	22.8	20.3	21.7	21.5	18.3–24.1%
		Day 5th	20.1	20	20.2	20.2	
19	PLT	Day 1st	172	76	128	332	125–618 × 10^9^/L
		Day 5th	144	122	18	113	
20	MPV	Day 1st	8.4	11.3	9.9	11.7	8.6–14.9 fL
		Day 5th	11.7	11.3	10.7	11.8	

WBC: white blood cells, LYM: lymphocytes, MON: monocytes, NEU: neutrophils, EOS: eosinophils, BAS: basophils, RBC: red blood cells, HGB: haemoglobin, HCT: haematocrit, MCV: mean corpuscular volume, MCH: mean corpuscular haemoglobin, MCHC: Mean corpuscular haemoglobin concentration, RDWc: Red blood cell distribution width, PLT: platelets, MPV: mean platelet volume.

**Table A3:** Comparison of haematological parameters pre and post Zarzio^®^ administration in cats from 2024.

No.	Parameter	Pre and Post Filgrastim Administration	CAT ID SAMPLE								
			33669	33008	32876	27478	32199	31846	31451	30918	Reference Range
1	WBC	Day 1st	1.08	0.21	0.34	0.77	0.37	2.26	0.6	2.45	3.5–20.7 × 10^9^/L
		Day 5th	5.64	5.94	19.32	31.07	20.27	12.35	7.13	20.94	
2	LYM	Day 1st	0.88	0	0.29	0.31	0.3	1.27	0.37	0.16	0.83–9.1 × 10^9^/L
		Day 5th	3.14	0.64	3.13	3.33	0.85	3.32	1.39	1.1	
3	MON	Day 1st	0.1	0	0.01	0.04	0.02	0.18	0.02	0.02	0.09–1.21 × 10^9^/L
		Day 5th	0.13	0.56	1.08	1.29	0.8	0.71	0.54	1.38	
4	NEU	Day 1st	0.08	0	0.04	0.37	0.05	0.74	0.11	1.96	1.63–13.37 × 10^9^/L
		Day 5th	2.31	4.69	14.83	26.11	18.45	8.25	5.13	18.38	
5	EOS	Day 1st	0.03	0	0	0.04	0	0.07	0.09	0.31	0.02–0.49 × 10^9^/L
		Day 5th	0.05	0.05	0.28	0.33	0.17	0.06	0.06	0.08	
6	BAS	Day 1st	0	0	0	0	0	0	0	0	0–0.2 × 10^9^/L
		Day 5th	0	0	0	0.01	0	0	0	0	
7	LYM%	Day 1st	81	0	86.5	40.6	82.2	56.3	62.2	6.6	0–100%
		Day 5th	55.7	10.8	16.2	10.7	4.2	26.9	19.6	5.2	
8	MON%	Day 1st	9.3	0	1.8	5.4	4.7	7.7	3.3	0.7	0–100%
		Day 5th	2.4	9.4	5.6	4.1	4	5.7	7.6	6.6	
9	NEU%	Day 1st	7.4	0	11.8	48.5	13.2	32.8	18.7	80	0–100%
		Day 5th	41	79	76.8	84	91	66.8	72	87.8	
10	EOS%	Day 1st	2.3	0	0	5.3	0	3.2	15.6	12.6	0–100%
		Day 5th	0.9	0.8	1.4	1.1	0.8	0.5	0.8	0.4	
11	BAS%	Day 1st	0	0	0	0.2	0	0.1	0.2	0.1	0–100%
		Day 5th	0	0	0	0	0	0	0	0	
12	RBC	Day 1st	6.6	8.43	8.15	8.64	9.19	8.72	7.85	6.25	7.7–12.8 × 10^12^/L
		Day 5th	6.87	7.43	7.55	8.26	6.98	8.38	5.92	6.99	
13	HGB	Day 1st	9.7	11.8	11.3	13.7	12.8	9.3	10.2	8.7	10–17 g/dL
		Day 5th	9.8	10.2	10.8	12.7	10.1	9.6	6.7	9	
14	HCT	Day 1st	29.97	37.29	36.02	39.36	40.21	32.01	33.29	27.9	33.7–55.4%
		Day 5th	30.86	32.51	33.72	38.83	31	30.79	23.74	31.29	
15	MCV	Day 1st	45	44	44	46	44	37	42	45	35–52 fL
		Day 5th	45	44	45	47	44	37	40	45	
16	MCH	Day 1st	14.7	14	13.9	15.9	13.9	10.7	13	14	10–16.9 pg
		Day 5th	14.2	13.7	14.3	15.4	14.5	11.4	11.4	12.9	
17	MCHC	Day 1st	32.4	31.6	31.4	34.8	31.8	29.1	30.7	31.4	27–35 g/dL
		Day 5th	31.7	31.4	32.1	32.6	32.7	31.1	28.3	28.9	
18	RDWc	Day 1st	23.2	25.2	23.3	22.9	25.5	22.7	21.8	22.6	18.3–24.1%
		Day 5th	23.6	25.2	22.5	22.5	24.7	21.6	21.3	22.6	
19	PLT	Day 1st	52	272	101	194	149	116	85	42	125–618 × 10^9^/L
		Day 5th	62	82	53	122	28	58	32	36	
20	MPV	Day 1st	14	10.9	13.1	14.3	12.6	9.5	11.7	9.9	8.6–14.9 fL
		Day 5th	12.4	12.1	13.9	14.1	12.7	9.8	10.7	11.3	

WBC: white blood cells, LYM: lymphocytes, MON: monocytes, NEU: neutrophils, EOS: eosinophils, BAS: basophils, RBC: red blood cells, HGB: haemoglobin, HCT: haematocrit, MCV: mean corpuscular volume, MCH: mean corpuscular haemoglobin, MCHC: Mean corpuscular haemoglobin concentration, RDWc: Red blood cell distribution width, PLT: platelets, MPV: mean platelet volume.

**Table A4:** CBC parameters comparison between pre (Day 1) and post (Day 5) Zarzio^®^ administration.

No.	Parameter	Day 1st			Day 5th			*p* Values	Reference Ranges
		Median Day 1st	Mean Day 1st	Standard Deviation Day 1st	Median Day 5th	Mean Day 5th	Standard Deviation Day 5th		
1	WBC	0.79	1.12	0.85	17.57	18.95	10.1	*p* < 0.01	3.5–20.7 × 10^9^/L
2	LYM	0.36	0.53	0.41	2.77	2.72	1.5	*p* < 0.01	0.83–9.1 × 10^9^/L
3	MON	0.02	0.09	0.23	0.84	0.90	0.57	*p* < 0.01	0.09–1.21 × 10^9^/L
4	NEU	0.11	0.46	0.72	13.94	15.07	8.75	*p* < 0.01	1.63–13.37 × 10^9^/L
5	EOS	0.01	0.04	0.07	0.15	0.18	0.14	*p* < 0.01	0.02–0.49 × 10^9^/L
6	BAS	0	0.00	0	0	0.00	0.01	*p* = 0.144	0–0.2 × 10^9^/L
7	RBC	8.42	8.69	1.74	6.98	6.80	1.65	*p* < 0.01	7.7–12.8 × 10^12^/L
8	HGB	11.65	12.15	2.64	9.45	9.27	2.54	*p* < 0.01	10–17 g/dL
9	HCT	34.76	37.10	6.69	30.82	29.25	7.49	*p* < 0.01	33.7–55.4%
10	MCV	44	41.7	6.57	44	42.90	2.63	*p* = 0.427	35–52 fL
11	MCH	13.95	14.03	1.34	13.6	13.58	1.42	*p* = 0.282	10–16.9 pg
12	MCHC	33	32.62	2.19	32.1	31.64	2.18	*p* = 0.144	27–35 g/dL
13	RDWc	21.95	22.06	1.5	20.65	21.28	1.58	*p* = 0.102	18.3–24.1%
14	PLT	145	167.45	98.52	56.5	70.04	51.4	*p* < 0.01	125–618 × 10^9^/L
15	MPV	11.65	11.49	1.49	11.75	11.44	1.61	*p* = 0.915	8.6–14.9 fL

WBC: white blood cells, LYM: lymphocytes, MON: monocytes, NEU: neutrophils, EOS: eosinophils, BAS: basophils, RBC: red blood cells, HGB: haemoglobin, HCT: haematocrit, MCV: mean corpuscular volume, MCH: mean corpuscular haemoglobin, MCHC: Mean corpuscular haemoglobin concentration, RDWc: Red blood cell distribution width, PLT: platelets, MPV: mean platelet volume.
